# Linear Space Requirements and Perch Use of Conventional Layer Hybrids and Dual-Purpose Hens in an Aviary System

**DOI:** 10.3389/fvets.2019.00231

**Published:** 2019-07-09

**Authors:** Mona F. Giersberg, Birgit Spindler, Nicole Kemper

**Affiliations:** ^1^Institute for Animal Hygiene, Animal Welfare and Farm Animal Behaviour, University of Veterinary Medicine Hannover, Hanover, Germany; ^2^Adaptation Physiology Group, Wageningen University and Research, Wageningen, Netherlands

**Keywords:** laying hen, roosting, aviary, image analysis, space requirement, welfare

## Abstract

Roosting on elevated perches is a behavioral priority in laying hens, which is well-investigated in both experimental and commercial settings. However, little is known about perching behavior and perch requirements of alternative hybrids, such as dual-purpose hens. The aim of the present study was to gain basic knowledge on linear space requirements and perching patterns of dual-purpose hens (Lohmann Dual, LD) by comparing them to a conventional layer line (Lohmann Brown plus, LB+). About 3,700 hens per genetic strain were housed in two consecutive batches in four compartments of an aviary system with metal perches at different heights above a grid tier. As an indicator for required perching space, the body widths of a sample of individual hens was determined by image analyses. In addition, the use of five differently located perches and one cross-brace (structural element of the aviary system) was assessed by photo-based observations during the light and the dark phase. The LD hens measured an average body width of 15.95 ± 0.08 cm, and thus occupied about 7% more linear space than the LB+ hens (14.77 ± 0.08 cm body width; *P* < 0.05). Overall perch use was higher during the dark compared to the light phase, both in the LB+ (3.89 ± 0.08 vs. 0.79 ± 0.03 hens/m, *P* < 0.05) and the LD hens (2.88 ± 0.06 vs. 0.86 ± 0.03 hens/m, *P* < 0.05). With a maximum of 8.17 hens/m, the LB+ hens preferred to roost on the highest perches available at night. In contrast, the LD hens also rested on the lowest perches, and showed a more even use of all perches provided. During the day, the LD hens seemed to need lower perches for easy access to the feeders, whereas more LB+ hens used the higher perches, presumably to avoid threatening conspecifics. The present results show that preferences for certain perch locations differed between conventional layers and dual-purpose hens, whereas diurnal patterns of perch use were similar in both hybrids. Therefore, perches should be designed and located in an aviary system to meet the specific preferences and behavioral needs of the hybrid housed.

## Introduction

The anti-predator response of roosting on elevated structures is still present in domestic laying hens, even if they are housed in indoor systems (1). In addition, commercial layer hybrids and their feral ancestors, the Jungle Fowl (*Gallus gallus*), show the same resting postures on perches (2). Since laying hens are willing to work to gain access to a perch at night (3), perching can be regarded as a behavioral priority (4). Furthermore, thwarting access to perches results in frustration (3), and may thus impair the birds' welfare. During daytime, the presence of perches reduces agonistic interactions among hens by increasing total space availability and decreasing bird density on the floor (5). Early access to perches has beneficial effects on the prevalence of non-aggressive abnormal behaviors, such as feather pecking and cloacal cannibalism, in later life (6, 7). In addition, feeding from perches was associated with less aggression and jostling at the feeder, longer undisturbed feeding bouts, and reduced mortality resulting from cannibalism (8).

Accordingly, the Council Directive 1999/74/EC laying down minimum standards for the protection of laying hens (9) demands perches of at least 15 cm length per hen, regardless of the housing system. However, no provisions are made regarding perch height, shape or material; although it is known that the acceptance and attractiveness of perches largely depend on these factors.

Previous work has shown that most hens chose the highest perch available for night-time roosting, both under experimental (3, 10) and observational conditions (11, 12). Particularly during the dark phase, laying hens seem to place a higher value on the height of a roosting site than on its surface design: more animals were found on high grids compared to low perches, although they clearly preferred perches to grids when offered at the same height (10). In commercially available and commonly used aviary systems, the standard perches are round steel pipes (12, 13), mainly for constructional and hygienic reasons. These perches are usually provided at different heights. However, similar to the Council Directive (9), other features, such as the hens' preferred perch diameter, shape or material, are not considered.

The propensity to perch is not only influenced by time of day and perch or facility design but also varies among chicken strains. Although selection processes seem to have not altered the motivation of perch use *per se*, perching behavior in fast growing broiler breeders appears to be restrained to some extent by their heavy body mass (14). However, variations in perch use were also observed among different layer strains (13). Brown lines, for instance were similar to each other and used the various tiers and roosting sites of an aviary system evenly. In contrast, two white layer strains showed more variation to one another, though both preferred the upper tiers of the aviary (13).

When comparing perch use between hybrid lines, it is important to consider the horizontal space occupied by the birds' bodies, i.e., their body widths. Birds from certain genetic strains may show a larger body width than other hybrids, and thus require a larger amount of perching space. By means of biometric measurements, it was shown that the body widths of brown pullets (Lohmann Brown and Lohmann Tradition) were significantly larger than those of white pullets (Lohmann Selected Leghorn) at the end of the rearing period (15). Similarly, adult Lohmann Brown laying hens required more linear space compared to Lohmann Selected Leghorn hens at any stage of production (16). By using a slightly different method to measure the body widths of four layer hybrid lines (Hy-Line Brown, Bovans Brown, DeKalb White, and Hy-Line W36) while perching spontaneously in their home pens, Riddle et al. (17) obtained similar results. The brown hybrids occupied significantly more perching space than the white hybrids, whereas the body widths did not differ between the two brown lines and the two white lines (17). Furthermore, body weight was not a reliable indicator for body width, neither in pullets nor in adult hens (15–17).

However, little is known about perch use, perch preferences or linear space requirements of dual-purpose hybrids. Dual-purpose lines, with hens laying a sufficient number of eggs and roosters showing an acceptable fattening performance, are kept as one alternative to the killing of male day-old chickens (18). The practice of killing newly hatched chickens raises socio-ethical concerns, and at present, it is also discussed whether it can be justified legally (18). Surveys of the Dutch public, for instance, showed that the participants were willing to pay a premium for poultry products if necessary to prevent the killing of male layer chickens (19). When asked to rank possible alternatives, the participants preferred “sex determination in the egg before incubation” and “keeping dual-purpose chickens” (19). However, there is evidence that the behavior of dual-purpose hens and high yielding layers differs in several aspects, and that commercial aviary systems should therefore be adjusted to the needs of the respective hybrid line (20). It was shown that young male dual-purpose chickens and male layer hybrids used elevated structures to a similar extent, and more frequently than fast growing broiler chickens (21). However, dual-purpose and broiler chickens preferred grids over perches at all daytimes, whereas similar numbers of male layer hybrids were found on grids and perches at dusk and night (21).

The aim of the present study was to assess linear space requirements and perch use of dual-purpose hens (Lohmann Dual, LD) in a commercial aviary system. This information can serve as a useful basis to adjust current housing systems to the behavioral needs of these hybrids. It was hypothesized that both the body width and the perching behavior of dual-purpose hens would differ compared to conventional layer hybrids (Lohmann Brown plus, LB+). Furthermore, it was expected that perch use within hybrid line would be affected by light phase and perch location in the aviary.

## Materials and Methods

All animals were housed according to EU (9) and national law (22, 23). In compliance with European Directive 2010/63/EU Article 1 5.v(f) (24), the present study did not imply any invasive procedure or treatment to the hens.

### Animals and Housing

**Figure 1:**
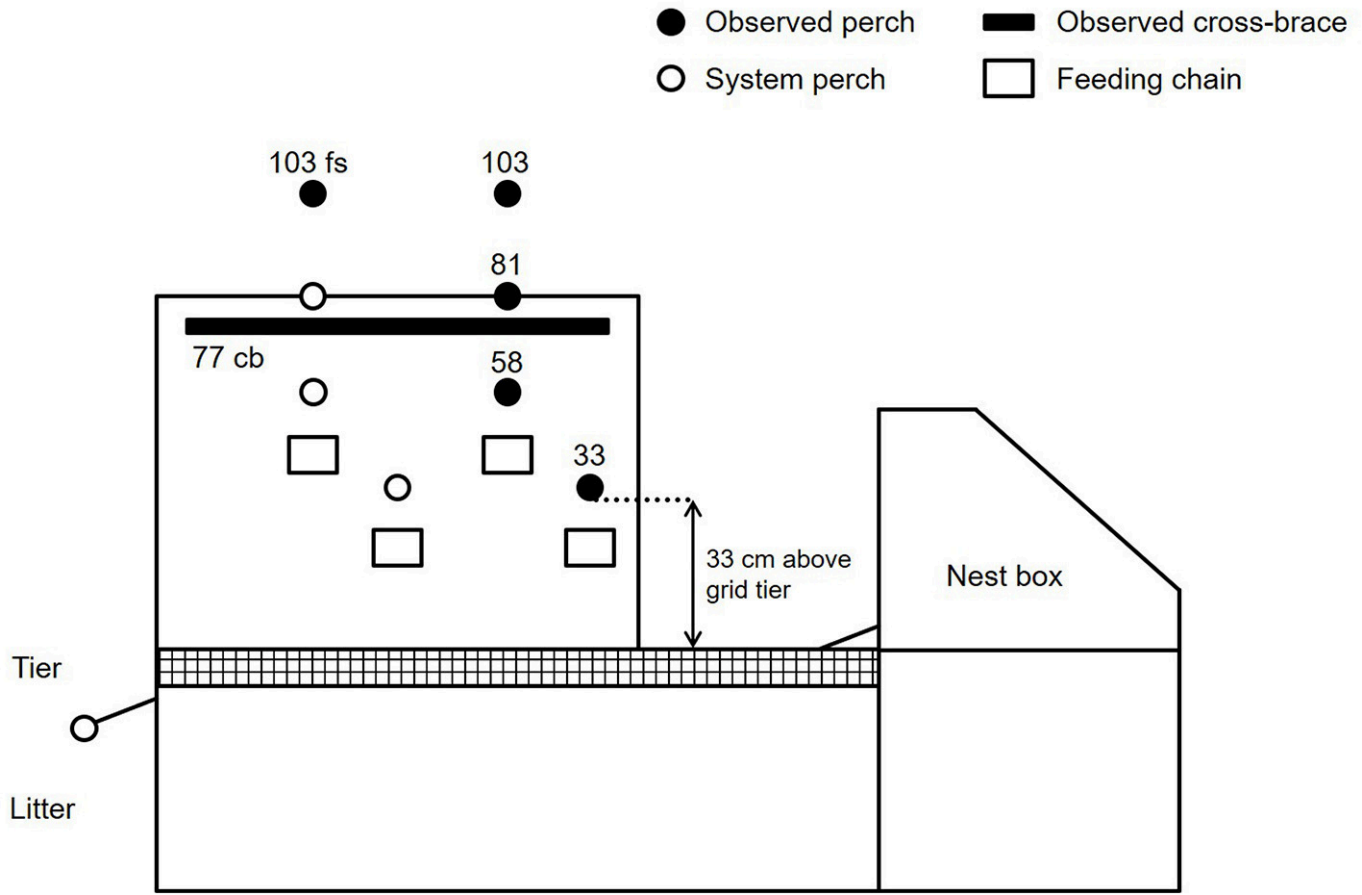
Cross section of the aviary system in which four perches (33, 58, 81, and 103) at the nest side, one perch (103 fs) at the floor side and one cross-brace (77 cb) were observed.

The study was carried out on the research farm “Ruthe” of the University of Veterinary Medicine, Hannover, Germany. Three-thousand-six-hundred-and-eighty-five Lohmann Brown plus (LB+, conventional layer hybrids) and 3,669 Lohmann Dual (LD, dual-purpose hybrids) hens with intact beaks were kept there in two batches from October 2015 to October 2016 (19th−71st week of life), and from November 2016 to November 2017 (18th−70th week of life), respectively. The two hybrid lines had the same housing conditions and standard management procedures (25), and the same setup with two stable compartments per line and batch (about 900 hens per compartment). Each compartment was equipped with six sections of an asymmetric aviary system (Natura Nova 270, Big Dutchman, Vechta, Germany). Each section of the aviary contained eight round metal perches (3.5 cm diameter) at four different heights above a grid tier (Figure 1) which offered about 17 cm perching space per hen. The total height of the aviary reached 200 cm above ground level. The light regime started with 10L:14D at 18/19 weeks of life and was gradually extended until 14L:10D (week 25). Alfalfa bales suspended in hay nets served as standard enrichment material (about 200 hens per bale). At first signs of feather pecking or cannibalism additional measures were taken following a graduated emergency scheme [for details see (26)]. Production parameters were recorded continuously by the farm manager.

### Determination of Body Width

The horizontal space occupied by the hens (= body width) was measured using a method described by Giersberg et al. (15). At 34 weeks of life, 110 LB+ and 104 LD hens with intact feather cover from the last batch were weighed individually (Manual poultry scale BAT1, VEIT electronics, Moravany, Czech Republic) and placed on a round metal perch (3.5 cm diameter) in a test cage located near the hens' home pens. The hens and a reference standard on the perch (10 cm length) were photographed from a front view using a digital camera (Olympus E-410, 17.5–45 mm lens, 10.0 megapixels, Olympus Optical Co GmbH, Hamburg, Germany) which was attached to a tripod in 160 cm distance to the test cage. The photographs were stored in a personal computer and analyzed with the software program ImageJ (version 1.52a). In each digital image an operator set a horizontal connecting line between the outer contours of the hen's wings at the level of the carpal joints. The carpal joints were chosen as marker points since they are clearly distinguishable and describe the widest points of the hen's body when depicted from a front view. In addition, this approach prevented the misleading inclusion of single protruding feathers in the measurements of the hen's body width. Based on the pixel count of the reference standard and its known length (in cm), and the pixel count of the connecting line, the program calculated the hen's body width (in cm) according to the rule of proportion. Since body position did not affect the linear space occupied (15), it was not taken into account whether the hen in the photo was standing or sitting on the test perch. Standing was defined as perching with legs stretched, whereas sitting meant that the hen's legs were more or less bent under the body. Sternal recumbency (i.e., the hen's sternum touching and its body protruding the perch) which may lead to distortions as the line between the carpal joints would be closer to the camera than the reference standard on the perch, did not occur. Photos in which the hens were not depicted from a front view but stood rather laterally on the perch were discarded prior to the measurements.

### Observations of Perch Use

Photo-based behavioral observations were carried out for two consecutive days per week at three times during the laying period (24th−27th, 38th−41th, and 54th−57th week of life). Therefore, a scouting camera (Snapshot Mini Black 5.0 MP, Dörr, Neu-Ulm, Germany) was installed in each stable compartment. The camera was mounted at an ~20° angle on a wire mesh element near the side wall recording the fourth section of the aviary system from the entrance of each compartment. Thus, it was ensured that the hens on all perches of interest were clearly visible in the pictures. The camera position was kept constant among compartments and batches. The observed perches are shown in Figure 1. Four of the perches were located at a height of 33, 58, 81, and 103 cm above the grid tier of the aviary at the nest side. In addition, a 103 cm high perch on the floor side (103 fs) and a 77 cm high rectangular shaped cross-brace of the aviary (77 cb), which was located at right angles to the perches, were observed. In total, 120 cm length per perch and 100 cm length of the cross-brace were recorded per compartment. On each observation day, the number of perching hens was detected for a total of 6.5 h during the dark phase (1.25 h before lights-on and 5.25 h after lights-off), and 9.5 h during the light phase (4.75 h after lights-on and 4.75 h before lights-off) via time sampling method (sample interval: 15 min). Due to the short durations of the twilight phases, and the resulting small numbers of sample points, data from these phases were assigned to the dark phase.

### Statistical Analysis

All statistical analyses were performed using the software SPSS Statistics (version 25, IBM, Armonk; NY, USA). Residuals were assessed visually for normality by creating histograms including the Gaussian distribution curve. Homogeneity of variance was tested according to the Levene procedure. Univariate analyses of variance were performed to tests hybrid effects on the hens' body weights and body widths. Data on perch use are presented in hens/m perch. The number of hens per meter perch did not differ between the two consecutive observation days per week, and neither among the sample points within the dark phase nor within the light phase. Therefore, data were combined to one value for the mean number of hens per meter perch for the dark and the light phase, for each week, each batch, each hybrid and stable compartment, and for each perch or the cross-brace, respectively. The values calculated in this way were then subjected to further statistical analyses. For an overview of the differences in perch use between the dark phase and the light phase, a generalized linear mixed model was calculated separately for both hybrids. Data were structured by observation period and week as repeated measures. The models consisted of the target variable hens/m perch (including the cross-brace), and the fixed effect light phase. Stable compartment and batch were added as random effects. Subsequently, generalized linear mixed models including observation period and week as repeated measures were used to test the effects of hybrid and perch type (including the cross-brace) on perch use during the dark and the light phase. The models consisted of the fixed effects hybrid and perch, and the random effects of stable compartment within hybrid and batch. All *post hoc* pairwise comparisons were adjusted by Bonferroni correction. Differences between the tested parameters were considered to be significant if *P*-values were <0.05. All data are presented as mean ± standard error of the mean (SEM).

## Results

### Production Data and Health Status

At the end of the laying period (week 70–71), the cumulative mortality was 9.49% in the LB+ flocks, and 5.10% in the LD flocks, respectively. Daily feed consumption per hen ranged between 97.57 g (LD) and 121.52 g (LB+). The laying rate was 84.88% (330 eggs/average hen housed) in LB+ hens, and 70.41% (274 eggs/average hen housed) in LD hens. During the entire production period, the health condition of the flocks was good. Antimicrobial or other veterinary treatment was not necessary. For detailed information on the feather and integument condition of the hens from the first batch see (27). Similar plumage and integument scores were observed in the LB+ and LD hens. from the second batch.

### Body Width of Conventional Layers and Dual-Purpose Hybrids

At 34 weeks of life, the LB+ and LD hens weighed 1875.41 ± 9.62 g, and 1786.47 ± 16.31 g, respectively. With an average body width of 15.95 ± 0.08 cm, the LD hens occupied about 7% (1.18 cm) more horizontal space than the LB+ hens (14.77 ± 0.08 cm body width). Hybrid effects were found for both the hens' body weight (F_1,212_ = 22.64, *P* < 0.05), and body width (F_1,212_ = 103.26, *P* < 0.05). Based on these measurements, a maximum of 6.77 LB+ hens and 6.27 LD hens could theoretically rest per meter perch length without touching a conspecific.

### Perch Use

In the LB+ hens, overall perch use was higher at night (dark phase) than during the light phase (F_1,573_ = 681.35, *P* < 0.05). The mean number of LB+ hens/m on all observed perches was 3.89 ± 0.08 during the dark, and 0.79 ± 0.03 during the light phase. Similar results were obtained for the LD hens. With 2.88 ± 0.06 hens/m, more LD hens were found on the perches at night than during the light phase (0.86 ± 0.03 hens/m; F_1,574_ = 287.03, *P* < 0.05).

#### Perch Use During the Dark Phase

**Figure 2:**
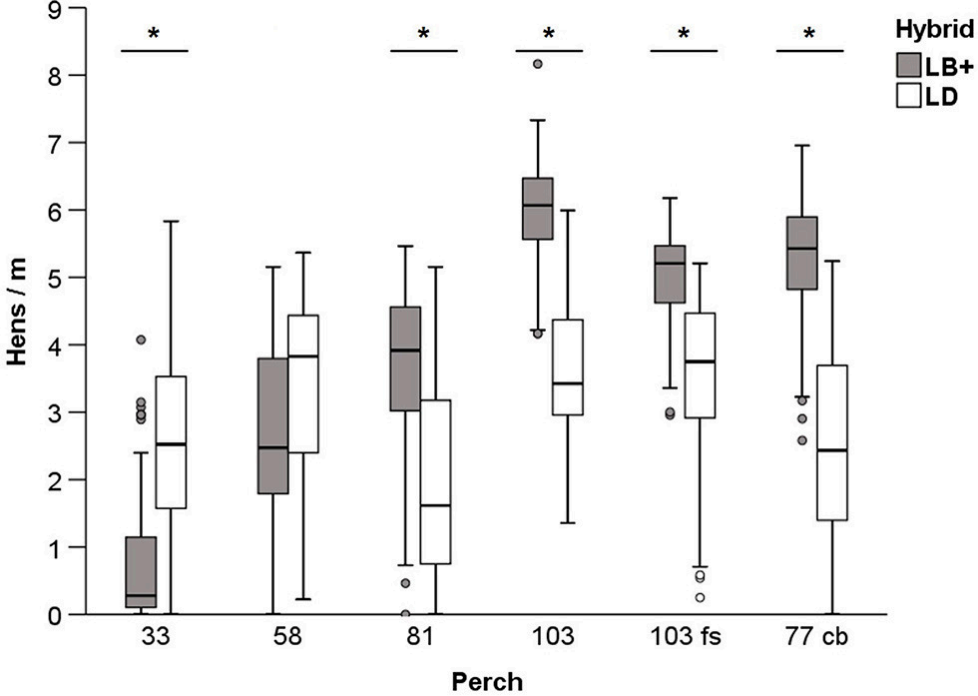
Perch use during the dark phase. Mean number of conventional layers (LB+) and dual-purpose (LD) hens/m ± SEM on different perches (33, 58, 81, 103, and 103 fs) and one cross-brace (77 cb). ^*^show significant differences (*P* < 0.05) between hybrids.

At night, the use of all observed perches was affected by hybrid (F_1,564_ = 9.53, *P* < 0.05; Figure 2). A higher number of LD hens was found on the lowest perche 33 (33 cm above the grid tier) compared to the LB+ hens. Perch 58 tended to be used by a larger number of LD hens (F_1,564_ = 3.43, *P* = 0.06). In contrast, more LB+ than LD hens rested on the higher perches 81, 103, 103 fs, and on the cross-brace (77 cb). In both hybrids, perch use was also affected by perch location (F_5,564_ = 121.60, *P* < 0.05; Table 1). Pairwise comparisons in the LB+ hens showed that the 103 cm high perch at the nest side of the aviary (103; 5.97 ± 0.08 hens/m) was preferred over all observed perches (*P* < 0.05). On this perch, a maximum number of 8.17 hens/m was found. The cross-brace (77 cb) and perch types 103 fs, 81, 58, and 33 followed in descending order, in which only the numbers of hens on the cross-brace (77 cb) and perch 103 fs did not differ (5.29 ± 0.09 and 5.00 ± 0.07 hens/m, respectively, *P* > 0.05). The LD hens occupied the perches 103, 103 fs and 58 to a similar extent (3.56 ± 0.11, 3.49 ± 0.13 and 3.36 ± 0.13 hens/m, respectively, *P* > 0.05), with a maximum of 5.99 hens/m observed on perch 103 (Table 1). Perch types 33 and 81, and the cross-brace (77 cb) were less frequented (*P* < 0.05), with no difference found between perch 33 and the cross-brace (77 cb) (*P* > 0.05).

**Table 1:** Perch use of conventional layers (LB+) and dual-purpose hens (LD) during the dark phase.

**Perch**						
	**33**	**58**	**81**	**103**	**103 fs**	**77 cb**
LB+	0.78 ± 0.09^a^	2.67 ± 0.13^b^	3.62 ± 0.13^c^	5.97 ± 0.08^d^	5.00 ± 0.07^e^	5.29 ± 0.09^e^
	(0.00–4.07)	(0.00–5.16)	(0.00–5.46)	(4.17–8.17)	(2.96–6.17)	(1.56–6.96)
LD	2.54 ± 0.14^a^	3.36 ± 0.13^b^	1.90 ± 0.15^c^	3.56 ± 0.11^b^	3.49 ± 0.13^b^	2.46 ± 0.15^a^
	(0.00–5.83)	(0.22–5.36)	(0.00–5.16)	(1.35–5.99)	(0.25–5.21)	(0.00–5.24)

Means in the same row with unlike superscripts differ significantly (P < 0.05).

*Mean numbers of hens/m ± SEM (minimum–maximum) on different types of perches (33, 58, 81, 103, and 103 fs) and on a cross-brace (structural element of the aviary, 77 cb)*.

#### Perch Use During the Light Phase

**Figure 3:**
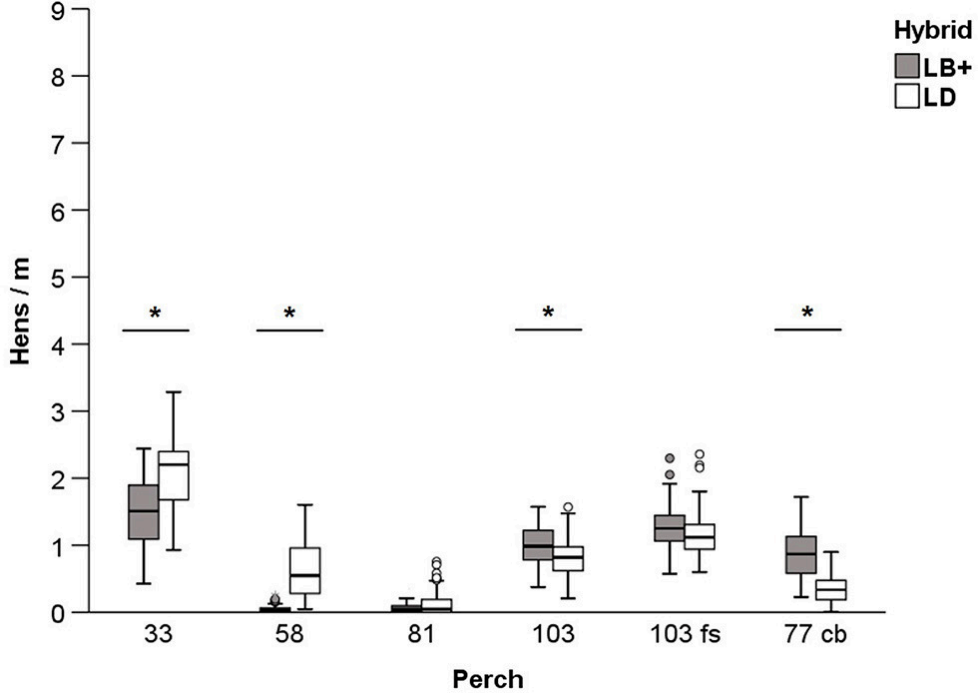
Perch use during the light phase. Mean number of conventional layers (LB+) and dual-purpose (LD) hens/m ± SEM on different perches (33, 58, 81, 103, and 103 fs) and one cross-brace (77 cb). ^*^Show significant differences (*P* < 0.05) between hybrids.

Hybrid effects on perch use during the light phase are presented in Figure 3. Similar to the observations at night, a higher number of the LD compared to the LB+ hens was found on the two lowest perches (33 and 58). However, more LB+ than LD hens were found on perch 103 and the cross-brace (77 cb) (*P* < 0.05). On perch types 81 and 103 fs, the number of observed hens/m did not differ between hybrids (F_1,563_ = 0.79, *P* = 0.37; F_1,563_ = 3.18, *P* = 0.06, respectively). Perch location affected perch use in both hybrids at day-time (F_5,563_ = 384.95, *P* < 0.05; Table 2). Perch 33 was the most frequented perch in the LB+ flocks (1.47 ± 0.05 hens/m, maximum: 2.44 hens/m), followed by perch 103 fs (1.25 ± 0.03 hens/m). With 0.05 ± 0.01 and 0.06 ± 0.01 hens/m, respectively, perch 58 and 81 were hardly used by the LB+ hens. In the LD flocks, pairwise comparisons showed that the number of hens/m differed between all of the observed perches (*P* < 0.05; Table 2). With an average of 2.08 ± 0.05 hens/m, and a maximum of 3.29 observed hens/m, the LD hens preferred the lowest perch (33) during the day. The perch least frequented by the LD hens was perch 81 (0.13 ± 0.02 hens/m).

**Table 2:** Perch use of conventional layers (LB+) and dual-purpose hens (LD) during the light phase.

**Perch**						
	**33**	**58**	**81**	**103**	**103 fs**	**77 cb**
LB+	1.47 ± 0.05^a^	0.05 ± 0.01^b^	0.06 ± 0.01^b^	1.00 ± 0.03^c^	1.25 ± 0.03^d^	0.89 ± 0.04^c^
	(0.42–2.44)	(0.00–0.26)	(0.00–0.40)	(0.38–1.57)	(0.57–2.30)	(0.23–1.72)
LD	2.08 ± 0.05^a^	0.62 ± 0.04^b^	0.13 ± 0.02^c^	0.82 ± 0.03^d^	1.15 ± 0.03^e^	0.35 ± 0.02^f^
	(0.93–3.29)	(0.05–1.60)	(0.00–0.75)	(0.20–1.57)	(0.60–2.36)	(0.00–0.90)

Means in the same row with unlike superscripts differ significantly (P < 0.05).

*Mean numbers of hens/m ± SEM (minimum–maximum) on different types of perches (33, 58, 81, 103, and 103 fs) and on a cross-brace (structural element of the aviary, 77 cb)*.

## Discussion

The objective of the present investigations was to determine linear space requirements and perch use of dual-purpose hens in a commercial aviary system. Therefore, the hens' body widths and the number of birds per meter perch served as indicators. As expected, dual-purpose hens (LD) differed in both aspects compared to a conventional layer strain (LB+). In general, the LD hens had larger body widths' and showed no clear preference to roost on the highest perch available at night. However, diurnal patterns of perch use were similar in the LB+ and LD hens.

Biometric measures provide useful information on the physical body dimensions of animals. The determination of a chicken's body width by digital image analyses is a suitable approach to define and discuss basic linear space requirements in poultry housing, such as minimum perching space (15). Previous investigations found no effects of age in adult laying hens (16) and body position on the linear space occupied (15, 16). Therefore, the body width measurements of the LB+ and LD hens were carried out at only one time during the laying period, and it was not taken into account whether the birds were standing or sitting on the test perch. As hypothesized, the birds' average body widths were affected by hybrid line. The LD hens occupied 7% more horizontal space, although they weighed about 5% less compared to the LB+ hens. However, “body width” measured as the horizontal line between the hen's carpal joints corresponds to a skeletal feature of the bird that is not necessarily related to its body weight (15). Consequently, Briese and Spindler (16) observed a significant decrease in the average body weight of an adult layer strain that was not accompanied by changes in the hens' body widths. In addition, Riddle et al. (17) found differences in the body widths of brown and white layer lines, although the body weights were similar among all four lines tested. With about 22 cm, their brown lines (Hy-Line Brown, Bovans Brown) showed larger body widths than the LB+ hens in the present study. This may be due to methodical differences, as Riddle et al. (17) measured the widest points of the bodies of birds perching in a sternal sitting position from top-view images (17). Furthermore, Hy-Line Brown and Bovans Brown hens may show a larger body frame size compared to LB+ hens, even though all lines were brown layer hybrids.

A typical increase in the number of hens using perches at nighttime has been reported across a variety of housing systems, such as small experimental pens (28), furnished cages (27), and commercial aviaries (11). In addition, this general pattern was consistent between different genetic lines, including white (11) and brown layer hybrids (27), and feral fowl (2). Brendler and Schrader (12) observed that perch occupancy in commercial aviary systems was about four times higher at night than during day. Correspondingly, a significantly larger number of both the LB+ and LD hens was detected on the elevated perches of the aviary system during the dark phase compared to the light phase. Therefore, LD hens seem to follow similar diurnal patterns of perching as conventional layer hybrids. Thus, the anti-predator hypothesis of roosting on elevated structures at night appears to be also valid for dual-purpose hens.

Hybrid effects were found for the use of nearly all observed perches during the dark phase. A larger number of LD hens roosted on the lowest perche (33 cm above a grid tier of the aviary) and a tendency was observed for the 58 cm high perch, whereas the higher perches (81–103 cm) were occupied to a larger extent by the LB+ hens. Strain differences in preferences for roosting sites in aviary systems were also reported by Ali et al. (13). Though overall perch use was higher in the brown layer lines, the white lines preferred the perch on the upper tier of the aviary. However, it is difficult to relate their findings to the present results, since they tested mere layer lines, and a multi-tier aviary system in which perches at different heights were not present on each tier. As the LD hens showed a lower average body weight than the LB+ hens, it is unlikely that access to the higher perches was restrained by their body mass, as it can be the case in fast growing broiler breeders (14). Similarly, it seems not likely that the available perch space was a limiting factor for the LD hens to roost on the upper perches, even though they had larger body widths than the LB+ hens. According to the present biometric measurements, about six LD hens could theoretically sit per meter perch without touching each other. In the current study, though, an average of less than four LD hens was found per meter perch, and the maximum number of hens/m did not exceed six hens at any observation point during nighttime. However, LD hens have a rather compact anatomy with relatively short legs due to a sex-linked dwarf gene (29). Thus, they might have encountered more difficulties to reach the higher perches. A further explanation might be that the LD hens experienced the proximity of conspecifics as aversive at densities of more than 4 hens/m, and thus used the total space provided by all the perches more evenly. Therefore, future research should also consider distribution indices, such as the hens' preferred NND (nearest-neighbor distances) when roosting on a perch (30).

Within the LB+ strain, perch use at night increased with increasing perch height. The highest average number of LB+ hens per meter was found on the perch located at 103 cm above the aviary tier. This is in line with former investigations (e.g., 3, 10, 11, and 12) in which laying hens showed a strong preference for the highest perches available. In the present study, a maximum of about 8 LB+ hens/m was observed on the 103 cm high perch. However, based on their body widths, slightly less than seven LB+ hens could sit per meter perch without touching a conspecific. Therefore, the hens huddled together, and seemed to accept being compressed by each other when resting on the highest perch. In an asymmetric aviary system, more hens were found on the perches at the floor side compared to those at the nest side, although their height above the aviary tier did not differ (12). The authors assumed that the hens might have perceived the total height of the perches at the floor side as higher, and therefore preferred them (12). In contrast, the LB+ hens used the highest perch at the nest side (type 103) to a larger extend compared to that at the floor side (103 fs). The LD hens showed no clear preference to roost on the highest perches. As mentioned above, this may be explained by their limited ability to access these perches, or by their possible preferences for less proximity while roosting. Particularly the low use of the perch at 81 cm height might be caused by the hens' difficulties to reach a certain roosting site, since it was mounted in a vertical line between the 58 cm and the 103 cm high perch.

Former studies indicate that laying hens may choose to rest on elements of the aviary system like cross-braces (31) or ledges (11), although sufficient total perch space is provided. Campbell et al. (11) found a considerable number of hens roosting on ledges, which were originally provided to assist the hens to move between the aviary tiers. Again, this was attributed to height preferences: due to a lack of perching space on the upper tiers of the aviary system, the hens chose to rest on the upper ledges rather than moving to proper perches on the less preferred lower tiers (11). In contrast, Giersberg et al. (31) observed a maximum number of hens on the cross-braces at any time of day, although sufficient perching space was offered at the same height. In the present study, the cross-brace (77 cb) was not the most preferred roosting site but it was frequently used by both hybrids. During the night, the LB+ hens occupied the cross-brace to a similar extent as the highest perch at the floor side (103 fs). In the LD hens, the use of the cross-brace was similar to that of the lowest perch (type 33). It is difficult to interpret why the cross-brace was preferred for instance over the slightly higher 81 cm perch. All observed elements of the aviary were made of metal; however, the cross-brace had a rectangular shape and was positioned at right angles to the longest side of the hen house, whereas the perches had a round profile and were arranged in parallel. Future research on roosting site preferences should therefore disentangle the effects of shape and arrangement of structural elements relative to proper perches.

During the light phase, the perches where used differently, which is in line with former investigations (12). However, hybrid effects were similar to those at night. Again, the two lower perches were used by a larger number of LD hens, whereas more LB+ hens were found on the highest perch at the floor side and the cross-brace. Within hybrid line, most LB+ hens were observed on the lowest perch, which provided access to a feeding trough. Since feeding from perches is associated with less aggression, less jostling and less disrupted feeding bouts (8), the LB+ hens may have chosen for this option instead of accessing a feeder from the aviary tier. However, since the current study focused on perch use, the feeding behavior and the numbers of hens feeding from the grid tier of the aviary system were not recorded. The second highest number of LB+ hens was found on the highest perch at the floor side (103 fs), followed by the highest perch at the nest side (type 103) and the cross-brace (77 cb), whereas the perches in a middle position (types 58 and 81) were hardly used. The LB+ hens, which showed injurious pecking (20, 26), may have accessed the higher perches during daytime to escape from threatening conspecifics (5), and thus to avoid being feather-pecked or cannibalized (6–8). Within the LD strain, the lowest perch was preferred during the light phase, and the average number of hens per meter was even higher compared to the LB+ flocks. Again, this may be explained by morphological differences between the two hybrid lines. For the compacter LD hens it might have been more difficult to reach a feeder from the aviary tier, whereas perching allowed easier access to a feeding through. Similar to perching patterns within the LB+ strain, the second and third most frequented perches were the highest ones at the floor side (103 fs) and at the nest side (type 103), although the LD hens showed no signs of behavioral deviations (22, 27). A possible explanation may be that the LD hens used the higher perches for undisturbed resting bouts during the day (12) instead of directly avoiding feather peckers. As in the dark phase, the perch 81 cm above the grid tier of the aviary was least used by the LD hens during the day.

The present study provides basic information on linear space requirements and perch use of dual-purpose hens in a commercial setting. It highlights the importance of taking into account the morphology and the specific perching preferences of the hybrid housed. Since dual-purpose hens show a larger body width compared to conventional layer hybrids, it must be ensured that they have sufficient perching space in existing housing systems. Furthermore, dual-purpose hens should be provided with perches at different heights on each tier of the aviary system, as they use the lower perches to access the feeders during the day as well as for roosting at nighttime. In order to further adjust aviary systems to the needs of dual-purpose hens, future research should elaborate on the hens' preferences for a certain perch shape or material. To test these preferences in detail, further indicators, such as time spent on the perch or perching frequency should be taken into account. Additionally, parameters such as keel bone and footpad status should be assessed, since the hens' choices might not always be the most beneficial for their health.

## Data Availability

The datasets for this study are available on request. The raw data supporting the conclusions of this manuscript will be made available by the authors (without undue reservation) to any qualified researcher.

## Ethics Statement

The experiments comply with the requirements of the ethical guidelines of the International Society of Applied Ethology (32). All animals were housed according to EU (9) and national law (22, 23). In compliance with European Directive 2010/63/EU Article 1 5.v(f) (24), the present study did not imply any invasive procedure or treatment to the hens. Therefore, after reading the proposal for permission to perform this study, the local Animal Research Authority (LAVES—Lower Saxony State Office for Consumer Protection and Food Safety) stated that no specific permission or approval by an ethics committee was required.

## Author Contributions

MG, BS, and NK designed the experiments. MG performed the data assessment, analyzed and interpreted the data, and wrote the paper. BS and NK helped to interpret the results and edited the manuscript. All authors read and approved the final manuscript.

### Conflict of Interest Statement

The authors declare that the research was conducted in the absence of any commercial or financial relationships that could be construed as a potential conflict of interest.
